# Phlebotomine sand fly fauna characterization and *Bartonella bacilliformis* DNA detection in *Pintomyia (Pifanomyia) robusta* at the Ecuador-Peru frontier

**DOI:** 10.1371/journal.pntd.0014288

**Published:** 2026-05-13

**Authors:** Victor O. Zorrilla, Andrés Carrazco-Montalvo, Liz J. Espada, Leonardo Fárez-Noblecilla, Marisa E. Lozano, Michael Kosoy, Clifton McKee, Craig A. Stoops, Ryan T. Larson, Renato León, Gissella M. Vásquez

**Affiliations:** 1 Department of Entomology, U.S. Naval Medical Research Unit SOUTH, Bellavista, Peru; 2 Laboratorio de Entomología Médica & Medicina Tropical LEMMT, Instituto de Microbiología, Colegio de Ciencias Biológicas y Ambientales COCIBA, Universidad San Francisco de Quito USFQ, Cumbayá, Quito, Ecuador; 3 Centro de Referencia Nacional de Genómica, Secuenciación y Bioinformática, Instituto Nacional de Investigación en Salud Pública “Leopoldo Izquieta Pérez”, Quito, Ecuador; 4 Culmen International LLC, Alexandria, Virginia, United States of America; 5 Laboratorio de Entomología 07D02, Machala-Salud, Ministerio de Salud Pública, Machala, El Oro, Ecuador; 6 KB One Health LLC, Fort Collins, Colorado, United States of America; 7 Department of Epidemiology, Johns Hopkins Bloomberg School of Public Health, Baltimore, Maryland, United States of America; Kenya Agricultural and Livestock Research Organization, KENYA

## Abstract

Phlebotomine sand flies are blood-sucking dipterans widely distributed in tropical and subtropical areas of the Americas and are important vectors of leishmaniasis caused by *Leishmania* spp. parasites and Carrion’s disease caused by the bacteria *Bartonella bacilliformis*. Both are a significant economic burden in rural areas and a major risk to military personnel deployed to endemic areas. To better understand transmission of these pathogens and epidemiological trends, sand flies were collected from nine sites across the Ecuador-Peru border region in 2015 and 2017 and screened for *Leishmania* using PCR targeting kinetoplast DNA, and *Bartonella* using PCR targeting the 16S-23S internal transcribed spacer (ITS) region, citrate synthase (*gltA*) gene, and NADH dehydrogenase subunit G (*nuoG*) gene. A total of 548 sand flies belonging to 15 species and 2,711 sand flies belonging to 11 species were collected in Ecuador and Peru, respectively. *Pintomyia (Pifanomyia) robusta* was generally the most abundant species found across sites sampled in Ecuador and Peru. In the Chinchipe River basin, *Pi. (Pif.) robusta*, *Pintomyia (Pifanomyia) maranonensis*, and *Lutzomyia (Helcocyrtomyia) castanea* were collected on both sides in Zamora-Chinchipe, Ecuador, and Namballe, Peru. Of the 637 phlebotomine sand fly pools screened, no *Leishmania* positives were found; however, nine pools of *Pi. (Pif.) robusta* collected in Ecuador were positive for *B. bacilliformis* based on phylogenetic analysis of the *gltA* gene. One *Pi. (Pif.) maranonensis* from Peru and one *Pi. (Pif.) robusta* from Ecuador were positive for *Bartonella* DNA sequences that were close to *Candidatus* Bartonella rondoniensis based on *gltA*. This is the first reported detection of *B. bacilliformis* DNA in *Pi. (Pif.) robusta*, providing evidence for the role of this sand fly species in transmission of this pathogen at the Ecuador-Peru border.

## Author summary

Carrion’s disease, caused by *Bartonella bacilliformis,* is endemic to the northern and central Peruvian Andean regions and some locations near the Ecuador-Peru border. While in Peru, the main phlebotomine sand fly vectors for *B. bacilliformis* have been characterized, no phlebotomine sand flies have been found infected with this bacterium on the Ecuadorian side, where Carrion’s disease has low prevalence. In this paper, the authors report for the first time the detection of *B. bacilliformis* DNA in *Pintomyia (Pifanomyia) robusta* from Ecuador. Although there is limited information on Carrion’s disease transmission on the Ecuadorian side, this finding indicates that phlebotomine sand fly vectors have a wide distribution along the Ecuador-Peru border. Phlebotomine sand fly surveillance and identification of putative *Bartonella* vectors across the Ecuador-Peru border is an important step towards the design of better control strategies and reducing the risk of transmission in this region.

## Introduction

Vector-borne diseases (VBDs) are a significant public health concern with complex transmission dynamics resulting from an intricate interplay between vectors, pathogens, animal reservoirs, and human populations [1,2]. Over 80% of the global population is affected by VBDs, contributing 17% of the estimated worldwide burden of infectious diseases, impacting the most economically disadvantaged communities and posing a significant risk to military personnel deployed to endemic areas [3]. In the Americas, leishmaniasis is a disease caused by at least 15 species of *Leishmania* parasites that affects over 37,000 people annually and impacts populations in 19 Latin America countries [4]. Carrion’s disease, caused by the bacterium *Bartonella bacilliformis*, is geographically restricted to the Andean regions of Peru, Ecuador, and Colombia [5], where it affects over 3,000 people annually [6].

At the Ecuador-Peru border, leishmaniasis transmission co-occurs with Carrion’s disease in southern Ecuador and northeastern Peru [7–10]. Both *Leishmania* parasites and *B. bacilliformis* are most likely transmitted by the same phlebotomine sand fly species despite the distinct epidemiology of these pathogens, which is related to multiple factors, including pathogen prevalence, the ecology of an area, phlebotomine sand fly behavior, and human activities [11,12]. In 2024, 897 cases of leishmaniasis were reported in Ecuador, 44 and 11 cases (6%) from Zamora-Chinchipe and Loja provinces, respectively, areas contiguous to the Peru border [13]. In Peru, 4,812 cases of leishmaniasis were reported in 2024, 954 (19.8%) from Cajamarca, Piura, and Amazonas states neighboring the Ecuador border [14]. In Ecuador, no cases of Carrion’s disease have been reported since 1996, except for two cases in 2022, reported as “*verruga peruana*”, a chronic cutaneous manifestation of Carrion’s disease [12]. Historically, Carrion’s disease has been an important neglected disease in Ecuador with reports dating from 1938 until 1996 [8,15,16], including a major outbreak reported in 1980 with more than 200 clinical cases of *verruga peruana* and five laboratory-confirmed cases from which *B. bacilliformis* was cultured [15]. In contrast, Carrion’s disease cases are reported regularly in Peru, with an important outbreak reported near the border with Ecuador in 2013–2014, including 428 cases reported from Piura state [17]. In 2024, 324 cases of Carrion’s disease were reported from Peru, 29 of those in Cajamarca state, located at the Ecuador-Peru border.

There is limited information on phlebotomine sand fly species implicated as vectors of *Leishmania* parasites and *B. bacilliformis* at the Ecuador-Peru border. In the 1990s, *Lutzomyia (Helcocyrtomyia) castanea*, *Pintomyia (Pifanomyia) maranonensis*, and *Pi. (Pif.) serrana* (later identified as *Pi. (Pif.) robusta*) were reported in Zumba, Zamora-Chinchipe province, Ecuador, but their role in pathogen transmission was not studied in detail [7]. Caceres *et al*. (1997) [10] reported *Pi. (Pif.) robusta* and *Pi. (Pif.) maranonensis* as potential vectors of Carrion’s disease in Peru’s northeast region based on epidemiological observations. Recently in Peru, *Pi. (Pif.) maranonensis* was found naturally infected with *B. bacilliformis* [18] and *Leishmania (Viannia) peruviana* [9]. The principal vectors of *Leishmania* and *B. bacilliformis* in Peru are *Lu. (Hel.) peruensis* and *Pi. (Pif.) verrucarum* [10]; however, these species have not been reported in Ecuador. In the Ecuadorian coastal region, species such as *Lutzomyia (Tricholateralis) gomezi*, *Nyssomyia trapidoi*, and *Lutzomyia (Helcocyrtomyia) hartmanni* have been implicated as potential vectors of *Leishmania (Viannia) panamensis* [19–21]. Records of phlebotomine sand fly species in the Peruvian coastal region are very limited. Because of the number of species involved and the dynamic nature of pathogen transmission, the assemblage of phlebotomine sand fly species playing a role in transmission of *B. bacilliformis* and *Leishmania* pathogens at the Ecuador-Peru border remains unclear, and characterizing phlebotomine sand fly species involved in transmission is critical for the development of strategies that reduce the burden of human bartonellosis and leishmaniasis. The goal of this study was to characterize the phlebotomine sand fly fauna by performing collections at endemic sites to identify potential vectors of *B. bacilliformis* and *Leishmania* parasites through molecular screening of specimens collected on both sides of the Ecuador-Peru border.

## Materials and methods

### Ethics statement

This study involved the collection and molecular analysis of phlebotomine sand flies and was approved as Non-Human Subject Research through the NAMRU SOUTH Institutional Review Board process (Project NAMRU6.2015.0018 “Co-occurrence of leishmaniasis and bartonellosis in the Peru-Ecuador frontier: characterization of sand fly vectors and potential reservoirs in rural villages and military outposts”).

### Phlebotomine sand fly surveillance

From July 2015 to January 2017, phlebotomine sand fly collections were conducted at sites near the Ecuador-Peru border where leishmaniasis and human bartonellosis have been reported. Sand flies were preserved for species identification; unfed female sand flies were further processed for molecular screening.

### Ecuador study sites

**Fig 1:**
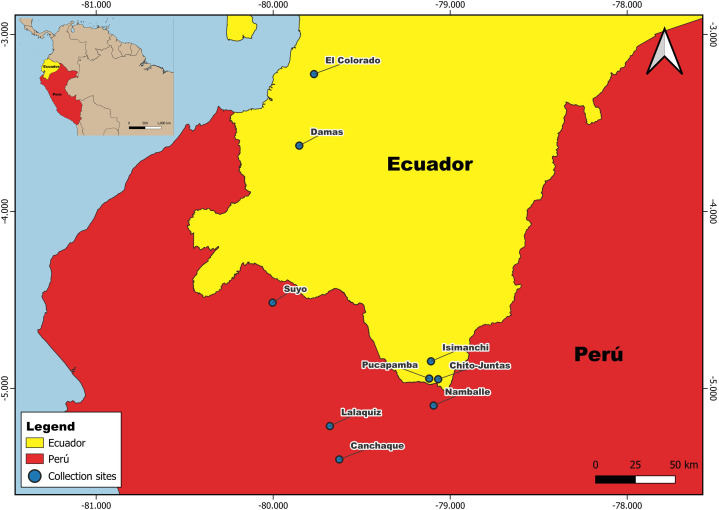
Study sites near the Ecuador-Peru border. Five study sites in El Oro and Zamora-Chinchipe provinces, Ecuador, and four study sites in Cajamarca and Piura states, Peru, were selected based on leishmaniasis and human bartonellosis case reports. The map was created in QGIS 3.40.13. Administrative boundaries were obtained from geoBoundaries (https://www.geoboundaries.org/) (Runfola et al. 2020) [22] and are provided under the Creative Commons Attribution 4.0 International (CC BY 4.0) license.

Phlebotomine sand fly collections were conducted at two study sites in El Oro (El Colorado/ 03°12’44.00“S, 79°45’53.7”W, 15 m.a.s.l; Damas/ 03°37’24.4”S, 79°50’52.2”W, 220 m.a.s.l), and three study sites in Zamora-Chinchipe (Isimanchi/ 04°50’50.6”S, 79°06’18.5”W, 1,287 m.a.s.l; Pucapamba/ 04°56’43.0”S, 79°07’03.8”W, 912 m.a.s.l; and Chito-Juntas/ 04°56’53.5”S, 79°04’02.6”W, 1,251 m.a.s.l) (Fig 1). El Oro is in the coastal region and has hills and plains extending into the Pacific Ocean. It has an average annual temperature ranging from 23 to 27 °C, with temperatures highest during the summer months (December-May) and cooler during the winter months (June-November). Zamora-Chinchipe is in the Andean region and is characterized by rugged mountainous relief. The average annual temperature ranges from 15 to 21 °C depending on altitude and location. Ecological information from collection sites was obtained from government sources [22,23].

### Peru study sites

Phlebotomine sand fly collections were conducted at four study sites along the Ecuador-Peru border: the rural community of San Pedro, Namballe district, Cajamarca state (05º05’48.3“S, 79º05’36.9”W, 1,197 m.a.s.l.) in the Peruvian northeastern region, and Lalaquiz (05º12’45.8”S, 79º40’47.1”W, 1,027 m.a.s.l.), Canchaque (05º24’03.8”S, 079º37’35.4”W, 838 m.a.s.l.), and Suyo (04°30’54.9”S, 80°00’11.8”W, 469 m.a.s.l.) districts, Piura state, in the western Andean region (Fig 1). A mountain chain separates both areas in a transitional zone between the high and western Andean foothills. The houses are dispersed in rural areas, surrounded by domestic crops and native forest. With the exception of Suyo district, coffee, cocoa, and fruit plantings are predominant in this region. The average annual temperature is over 25 ºC. Rainfall is abundant from January to March [24].

### Collection methods

**Fig 2:**
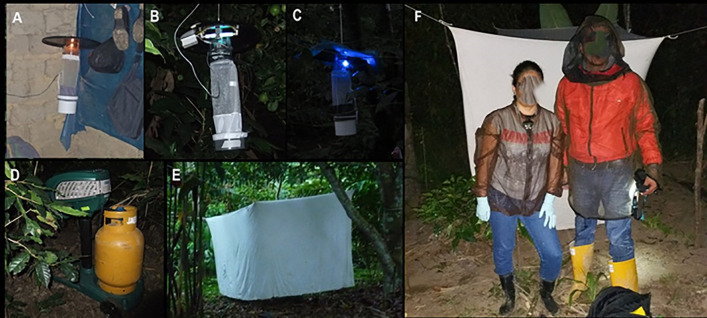
Trap types used for phlebotomine sand fly collections. **A)** Mini CDC light trap model 512; **B)** CDC blacklight UV trap model 1212; **C)** Mini CDC blue LED; **D)** Mosquito Magnet model Independence baited with CO_2_ and R-Octenol; **E)** Shannon trap; F) protected human landing collection (Photos: VZ).

Phlebotomine sand fly collection locations within each study site were selected based on the occurrence of leishmaniasis and bartonellosis cases reported by the local Ministry of Health. Collections were performed from July 2015 to January 2017 – totaling 30 days and 2,541 hours on the Peruvian side and 13 days and 552 hours on the Ecuadorian side. Collection equipment included Mini CDC light traps (CDC), blacklight UV traps (UV), Mini CDC light traps adapted with blue LED color bulbs (CDC Blue LED), Mosquito Magnet traps (MM) baited with CO_2_ and R-Octenol that operated from 1800 to 0600 hours and Shannon traps that operated from 1800 to 2100 hours), resting landing collections (1800–2100 hours and 0600–0800 hours), and protected human bait (1800–2200 hours) were also performed [20,25,26]. Table 1 summarizes the collection effort per site and collection method in both countries. Collections were carried out inside houses with Mini CDC light traps, CDC light traps adapted with blue LED, and resting collections, and outside houses with all collection methods mentioned above (Fig 2).

**Table 1:** Number of collections and total hours-trap for phlebotomine sand fly collections in Ecuador and Peru border during 2015-2017.

**Country**	**Locality**	**Ecological region**	**CDC**	**CDC-Blue LED**	**UV**	**Mosquito Magnet**	**Shannon**	**Resting**	**Protected Human Bait**	**Total**
**Ecuador**	Damas	Coastal	21			3				24
	El Colorado	Coastal	12							12
	Chito-Juntas	Andean region							3	3
	Isimanchi	Andean region	4							4
	Pucapamba	Andean region	2			3				5
**Peru**	Namballe	Andean region	69	27	4	13	4	2		119
	Lalaquiz	Western valley	24	12	2	5	1			44
	Canchaque	Western valley	30	15		6		6		57
	Suyo	Coastal	2					1		3
	**Total collections**		**164**	**54**	**6**	**30**	**5**	**9**	**3**	**271**
	**Collection effort (hours)**		**1968**	**648**	**72**	**360**	**15**	**18**	**12**	**3093**

### Phlebotomine sand fly taxonomic identification

Collected phlebotomine sand fly specimens were sorted by sex and stored in tubes with 70% ethanol in the field and transported to the Medical Entomology and Tropical Medicine Laboratory at Universidad San Francisco (LEMMT-USFQ) in Quito, Ecuador, or the Entomology Department at NAMRU SOUTH in Bellavista, Peru. Female specimens were processed, clarifying only the last abdominal segments and head for morphological identification; the rest of the body was pooled and preserved in 70% ethanol for molecular biology analysis [27]. Male specimens were clarified and permanently mounted in chloral gum or euparal [27,28], and morphological identification was performed using taxonomic keys by Galati (2014) [29] supported by the key of Young & Duncan (1994) [5]. The use of generic and subgeneric abbreviations was conducted following Marcondes (2007) [30].

### Data analysis

The Standardized Index of Species Abundance (SISA) was calculated in Excel 365 (Microsoft) using the Index of Species Abundance (ISA) to identify patterns of relative abundance of phlebotomine sand fly species across different habitats, regardless of the number of species collected [31,32]. Shannon (H) and evenness (E) indices of species diversity were also calculated [33]. The Shannon index (H) combines the number of species with the proportion of captured individuals of each of them using natural logarithms and will be affected by the number of species collected. The evenness index serves to understand if the community is more equitable in terms of species representation and is calculated based on the Shannon index divided by the total species richness (S). The Hutcheson t-test was used to compare Shannon indices among sites [33]. We quantified the number of phlebotomine sand fly specimens captured per hour per trap over each day of trapping and cumulatively over all trapping days for each study site. Since the data did not meet the normality requirement, a non-parametric Kruskal-Wallis test was performed to assess whether phlebotomine sand fly capture rates per hour varied significantly between trap types for each site. All analyses were carried out using the software PAST v4.

### Molecular screening of *Leishmania* and *Bartonella* DNA in sand flies

**Phlebotomine sand fly pooling.** Non-engorged phlebotomine sand fly females were pooled (1–10 specimens per tube) in microcentrifuge tubes with 70% ethanol, according to species identity, collection date, site, and trap type, labelled with a laboratory code, and stored at -30 ºC. A total of 182 pools (415 individuals) from Ecuador and 455 pools (1,355 individuals) from Peru were screened for pathogen detection. In Ecuador, 66 phlebotomine sand fly pools (150 specimens) from Chito-Juntas, 18 pools (27 specimens) from Pucapamba, 4 pools (4 specimens) from Isimanchi, 49 pools (81 specimens) from Damas, and 45 pools (153 specimens) from El Colorado were tested. In Peru, 251 phlebotomine sand fly pools (945 specimens) from Namballe, 124 pools (312 specimens) from Canchaque, 65 pools (75 specimens) from Lalaquiz, and 15 pools (23 specimens) from Suyo were tested.

**DNA extraction.** Genomic DNA of phlebotomine sand fly female pools was extracted using the DNeasy Blood & Tissue kit (QIAGEN, Valencia, CA, USA) according to the manufacturer’s protocol. DNA was eluted in 50 µL of elution buffer and frozen at -30 ºC.

**Molecular detection of *Leishmania* DNA.** Screening for *Leishmania* kinetoplast DNA (kDNA) was performed according to a PCR methodology previously described [34]. *Leishmania* (*Viannia*) *braziliensis* and *Leishmania* (*Leishmania*) *infantum* DNA were used as positive PCR controls, whereas PCR mix without DNA was used as a negative control.

**Molecular detection of *Bartonella* DNA.** The initial screening for *Bartonella* DNA was performed using PCR to amplify the 16S-23S internal transcribed spacer (ITS) [35]. To minimize locus-specific false negatives and improve overall detection sensitivity, nested PCR targeting the citrate synthase (*gltA*) gene [36–38] was performed on all pools, including ITS-negative samples. PCR targeting the NADH dehydrogenase gamma subunit (*nuoG*) [39] was also used for confirmation of positive samples from Ecuador. *B. bacilliformis* DNA from culture (SANDI strain NAMRU SOUTH, from a human case in Ancash, Peru) [38] was used as a positive control and PCR mix without DNA as a negative control. PCR products were visualized by 1.5% agarose gel electrophoresis in TAE buffer stained using SYBR Safe 10000X (Thermo Fisher Scientific, Waltham, MA, USA) at LEMMT Lab and Gel Red 10000X (Biotium, Inc., Fremont, CA, USA) at NAMRU SOUTH.

***Leishmania* and *Bartonella* DNA sequencing and phylogenetic analysis.** PCR amplicons were purified and sequenced using the BigDye Terminator v3.1 Cycle Sequencing kit (Applied Biosystems, Waltham, MA, USA) according to the manufacturer’s instructions. Only samples with PCR products that resulted in correct amplicon size and a clear gel electrophoresis band were considered positive. Positive samples were sequenced using the Sanger method at Macrogen, Seoul, South Korea (LEMMT-USFQ) or at the Entomology Laboratory at NAMRU SOUTH (Lima, Peru) using an Applied Biosystems 3130 XL Genetic Analyzer sequencer. Sequences were assembled and cleaned using Geneious Prime 2019.1.1. Sequences were compared with other samples belonging to *B. bacilliformis* and other *Bartonella* reference sequences using the BLASTn tool from the National Center for Biotechnology Information (NCBI; http://blast.ncbi.nlm.nih.gov/Blast.cgi; accessed on 26 October 2022). The *gltA* reference sequence for *Candidatus* Bartonella rondoniensis is not available via NCBI and was obtained via personal communication (C. McKee) with the authors of the original study that reported this lineage [40].

Phylogenetic analyses were performed to compare the Peruvian and Ecuadorian sequences positive for *Bartonella* DNA with other *Bartonella* species and sequences from recent studies on *Bartonella* in sand flies in Peru [25], Mexico [41], and Brazil [42,43]. Sequences of each gene with positive detections were aligned using MAFFT v7 with the local, iterative L-INS-i method [44] for the *gltA* and *nuoG* genes and the global, iterative G-INS-i method for ITS. Sequences from the *gltA* and *nuoG* genes were trimmed to equal length using trimAl [45], while ITS sequences were trimmed manually. Simultaneous phylogenetic model selection and maximum likelihood phylogenetic inference were performed using IQ-TREE v2.1.3 [46]. Phylogenetic trees were prepared using the *ggtree* package in R v4.3.0 [47,48].

## Results

### Entomological collections in Ecuador

A total of 548 phlebotomine sand flies (487 females and 61 males) were collected at five study sites located in El Oro and Zamora-Chinchipe provinces near the Ecuador-Peru border during 2015–2017. Seven genera and 15 species were identified, and species composition varied by location (Table 2). In Damas and El Colorado, El Oro province, *Pressatia* spp. (23.9%), *Nyssomyia trapidoi* (14.1%), *Pr. dysponeta* (12.8%), and *Lutzomyia (Tricholateralis) gomezi* (7.3%) were the most abundant phlebotomine sand fly species collected. *Psathyromyia (Psathyromyia) shannoni* and *Pi. (Pif.) serrana,* potential leishmaniasis vectors, were collected only in Damas; other non-vector species were collected at lower abundance in El Colorado. In Isimanchi, Pucapamba, and Chito-Juntas, Zamora-Chinchipe province, only three phlebotomine sand fly species were collected, with *Pi. (Pif.) robusta* (34.1%) being the most abundant across sites, particularly in Chito-Juntas (Table 2). *Pressatia* spp. was most frequently collected outdoors (64.9%) in Damas and El Colorado, whereas *Pr. dysponeta* were more abundant in the forest (80.0%) particularly in El Colorado; *Pi. (Pif.) robusta* was most frequently collected in the forest (78.6%) especially in Chito-Juntas (S1 Table).

**Table 2:** Phlebotomine sand fly species (females and males) collected and identified in five study sites from Ecuador during 2015-2017.

Species	Damas		El Colorado		Isimanchi		Pucapamba		Chito-Juntas		Total		Grand	%	SISA
	**F**	**M**	**F**	**M**	**F**	**M**	**F**	**M**	**F**	**M**	**F**	**M**	**Total**		
*Pi. (Pif.) robusta*					4	7	29		147		180	7	187	34.1	0.600
*Pi. (Pif.) maranonensis*					2		1		3		6		6	1.1	0.533
*Pressatia* spp.	17	3	111								128	3	131	23.9	0.378
*Ny. trapidoi*	51	20	6								57	20	77	14.1	0.333
*Pr. dysponeta*		14	56								56	14	70	12.8	0.311
*Lu. (Trl.) gomezi*	4	1	35								39	1	40	7.3	0.256
*Mg. (Bla.) gorbitzi*		15										15	15	2.7	0.156
*Lu. (Hel.) castanea*									1		1		1	0.2	0.156
*Lu. (Hel.) hartmanni*	4		1								5		5	0.9	0.122
*Pa. (For.) barrettoi majuscula*		2								2		2	0.4	0.111	
*Pa. (Psa). abonnenci*	5										5		5	0.9	0.100
*Ps. amazonensis*	2		1								3		3	0.5	0.078
*Pa. (For.) aragaoi*			1								1		1	0.2	0.056
*Pa. (Psa.) undulata*			1								1		1	0.2	0.056
*Pi. (Pif.) serrana*	2										2		2	0.4	0.022
*Pa. (Psa.) shannoni*	1	1									1	1	2	0.4	0.022
**Total**	**86**	**54**	**214**		**6**	**7**	**30**		**151**		**487**	**61**	**548**	**100.0**	
**Percentage**	**25.6%**	**39.1%**	**2.4%**	**5.5%**	**27.6%**										

Most specimens were collected with Mini CDC light traps (63.9%), but a substantial number were collected with protected human bait (27.6%) in a secondary forest close to Mayo River and adjacent to a gold mining area in Chito-Juntas. *Pintomyia (Pif.) robusta* was the most abundant species collected with protected human bait (147/151, 97.4%) (S2 Table). The average number of female and male phlebotomine sand flies collected hourly per trap by species is shown in the S3 Table. A Kruskal-Wallis test showed no significant differences in the number of phlebotomine sand flies collected hourly or daily among different trap types at each site (chi-squared = 2.33, df = 6, p > 0.05), despite the variation in phlebotomine sand fly trapping rates for the same trap type across sites.

### Entomological collections in Peru

A total of 2,711 phlebotomine sand flies (1,698 females and 1,013 males) were collected at four study sites during 2015–2016, belonging to 7 genera and 11 species. The distribution of phlebotomine sand fly species varied by location (Table 3). In the western valleys of Canchaque, Lalaquiz, and Suyo (Piura state), a total of 11 phlebotomine sand fly species were collected and identified: *Micropygomyia (Micropygomyia)* spp. (13.6%), *Lutzomyia (Helcocyrtomyia) ayacuchensis* (5.8%), *Pa. (Psa.) shannoni* (4.8%), and *Warileya lumbrerasi* (0.4%) were collected mostly in Canchaque but also in Lalaquiz; *Micropygomyia (Micropygomyia) cayennensis cayennensis* (3.7%), *Evandromyia (Barretomyia) sallesi* (1.3%), and *Lu. (Trl.) gomezi* (0.3%) were collected at all three sites, while *Psychodopygus panamensis* (0.2%) and *Pi. (Pif.) verrucarum* (0.04%) were collected only in Lalaquiz district. In Namballe district, Cajamarca state, three phlebotomine sand fly species, *Pi. (Pif.) robusta* (36.5%) *Pi. (Pif.) maranonensis* (17.8%), and *Lu. (Hel.) castanea* (15.6%) were collected, yet lower numbers of these species were recorded in Piura state (Table 3).

**Table 3:** Phlebotomine sand fly species (females and males) collected and identified in four study sites from Peru during 2015-2016.

Species	Namballe		Canchaque		Lalaquiz		Suyo		Total		Grand	%	SISA
	**F**	**M**	**F**	**M**	**F**	**M**	**F**	**M**	**F**	**M**	**Total**		
*Mi. (Mic.) cayennensis cayennensis*			19	29	7	10	26	8	52	47	99	3.7	0.641
*Pi. (Pif.) robusta*	647	319	5	4	7	7			659	330	989	36.5	0.554
*Mi. (Micropygomyia)* spp.			81	223	32	33			113	256	369	13.6	0.500
*Ev. (Bar.) sallesi*			19	12	2	1	2		23	13	36	1.3	0.467
*Lu. (Hel.) castanea*	274	132	1		15				290	132	422	15.6	0.457
*Pa. (Psa.) shannoni*			72	28	10	19			82	47	129	4.8	0.435
*Lu. (Hel.) ayacuchensis*			144	6	7				151	6	157	5.8	0.370
*Lu. (Trl.) gomezi*			1	5	1	1	1		3	6	9	0.3	0.348
*Pi. (Pif.) maranonensis*	309	172			0	1			309	173	482	17.8	0.250
*Warileya lumbrerasi*			7	1	4				11	1	12	0.4	0.228
*Lu. (Lutzomyia)* spp.							1		1		1	0.0	0.196
*Ps. panamensis*					3	1			3	1	4	0.1	0.109
*Pi. (Pif.)* species group *Verrucarum*			1						1		1	0.0	0.065
*Pi. (Pif.) verrucarum*					0	1			0	1	1	0.0	0.022
Total	1230	623	350	308	88	74	30	8	1698	1013	2711	100.0	
Percentage	68.4%		24.3%		6.0%		1.4%						

*Pintomyia (Pif.) robusta* was the most abundant species collected both indoors (53.6%) and outdoors (46.4%). In contrast, the other phlebotomine sand fly species displayed peri- and extra-domiciliary behavior and were collected in resting places such as in sheep, pig, and goat pens; chicken coops; bases of trees; rock crevices; house walls; coffee, cacao, and fruit tree plantings (S4 Table). Most phlebotomine sand fly specimens were collected with Mosquito Magnet (42.57%), followed by Mini CDC light traps (31.43%) (S5 Table). The average of phlebotomine sand fly species collected per hour/trap is shown in the S6 Table. A Kruskal-Wallis test showed significant differences in the number of phlebotomine sand flies collected hourly or daily per trap type per hour/day across sites (chi-squared = 51.41, df = 23, p = 0.0012). Phlebotomine sand fly collections with the Mosquito Magnet were performed outdoors near coffee, cocoa, and fruit crops.

### Diversity indices and abundance of phlebotomine sand fly species

#### Ecuador.

**Fig 3:**
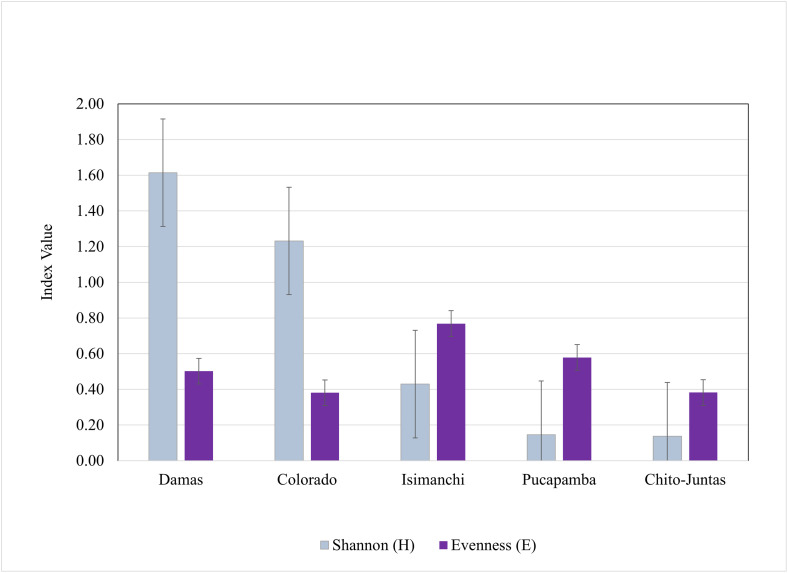
Shannon and evenness indices of phlebotomine sand fly species collected from July 2015 to January 2017 in study sites in El Oro and Zamora-Chinchipe provinces, Ecuador.

The Standardized Index of Species Abundance (SISA) estimates for Ecuadorian phlebotomine sand fly species indicated that *Pi. (Pif.) robusta* (SISA = 0.600), *Pi. (Pif.) maranonensis* (SISA = 0.533), *Pressatia* spp. (SISA = 0.378), *Ny. trapidoi* (SISA = 0.333), and *Pr. dysponeta* (SISA = 0.311) were the most abundant across all collection sites (Table 2). *Pi. (Pif.) robusta* (SISA = 0.908), *Ny. trapidoi* (SISA = 0.598), and *Pi. (Pif.) maranonensis* (SISA = 0.460) were the most predominantly collected species across all collection methods (S2 Table). Shannon and evenness indices were also analyzed by study site (Fig 3), with higher diversity in El Colorado (H = 1.23) and Damas (H = 1.61) in El Oro province, than in Isimanchi (H = 0.43), Pucapamba, (H = 0.15), and in Chito-Juntas (H = 0.14) in Zamora-Chinchipe province. While Isimanchi had the highest evenness index (E = 0.77), it also had the lowest number of phlebotomine sand fly collections across the sites. Diversity indices differed significantly across sites (Hutcheson t-test, p < 0.001).

#### Peru.

*Micropygomyia* (*Mic.*) *cayennensis cayennensis* (SISA = 0.641), *Pi.* (*Pif.*) *robusta* (SISA = 0.554), and *Mi. (Micropygomyia)* spp. (SISA = 0.500) were the most frequently collected species across all collection sites (Table 3). *Pi.* (*Pif.) robusta* (SISA = 0.840), *Lu. (Hel.) castanea* (SISA = 0.800), and *Pi. (Pif.) maranonensis* (SISA = 0.767) were the most frequently collected species across all methods used. The Mosquito Magnet trap collected the highest number of individuals of any method, capturing 42.6% of the total specimens collected (S5 Table).

**Fig 4:**
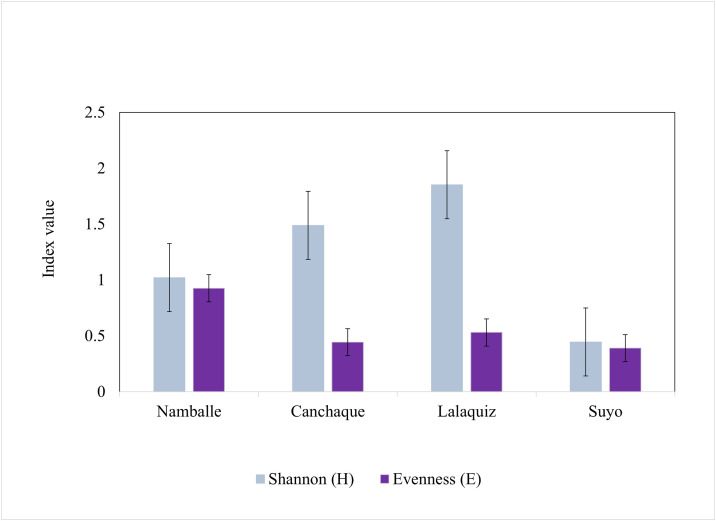
Shannon and evenness indices of phlebotomine sand fly species collected from July 2015 to August 2016 in study sites in Cajamarca and Piura states, Peru.

In Lalaquiz (11 species) and Canchaque (8 species), Piura state, the Shannon index was higher (H = 1.85 and 1.49, respectively) compared to Namballe (H = 1.02) and Suyo (H = 0.45). In Namballe, Cajamarca state, where only three species were identified, the evenness index (E = 0.93) showed that the frequency of these species was more equitable compared to Lalaquiz (E = 0.53), Canchaque (E = 0.44), and Suyo (E = 0.39) (Fig 4). The Shannon index differed across sites (Hutcheson t-test, p < 0.001).

### Molecular screening

A total of 1,770 sand flies were grouped in 637 pools and screened for *Leishmania* and *Bartonella* DNA by PCR. All phlebotomine sand fly pools were negative for *Leishmania* DNA by kDNA PCR. Two phlebotomine sand fly pools of *Pi. (Pif.) robusta* from Chito-Juntas, Ecuador, were positive for *Bartonella* DNA by *nuoG* and ITS PCR, and ten phlebotomine sand fly pools (4 pools from Chito-Juntas, 1 from Isimanchi, and 5 from Pucapamba) were positive for *Bartonella* DNA by nested *gltA* PCR (Table 4). *Pintomyia (Pif.) robusta* specimens positive for *Bartonella* DNA were collected indoors with CDC light traps in Isimanchi and Pucapamba (2 specimens), outdoors with Mosquito Magnet in Pucapamba (4 specimens), and in the secondary forest with protected human bait in Chito-Juntas (4 specimens). Positive controls were successfully amplified in all reactions. *Bartonella* PCR tests of sand flies from Peru detected four pools of Namballe sand flies positive by ITS PCR and confirmed by nested *gltA* PCR: *Pintomyia (Pif.) maranonensis* (two pools), *Pi. (Pif.) robusta* (one pool), and *Lu. (Hel.) castanea* (one pool). *Bartonella* positive-*Pi. (Pif.) maranonensis* were collected with a Mosquito Magnet trap outside a house on the forest edge. The minimum *Bartonella* spp*.* infection rate was 0.3%, based on positive *gltA* PCR, which was the most sensitive marker for these samples.

**Table 4:** Phlebotomine sand fly pools positive for *Bartonella* DNA by study site, trap type, and molecular marker.

Country	Study site	Trap type	Collection site	Sandfly/hour/trap	Sample ID	Sand fly species	ITS PCR	*nuoG* PCR	*gltA* PCR
Peru	Namballe	Mosquito Magnet	Forest edge	0.42	PEC15–170	*Pi. (Pif.) maranonensis*	+		+
Ecuador	Chito-Juntas	Protected human bait	Secondary forest	0.25	A18_PEC	*Pi. (Pif.) robusta*			+
Ecuador	Chito-Juntas	Protected human bait	Secondary forest	0.25	A39_PEC	*Pi. (Pif.) robusta*	+	+	+
Ecuador	Pucapamba	Mini CDC light trap	Intradomiciliary	0.08	2-70_PEC	*Pi. (Pif.) robusta*			+
Ecuador	Pucapamba	Mosquito Magnet	Peridomiciliary	0.08	2-68_PEC	*Pi. (Pif.) robusta*			+
Ecuador	Pucapamba	Mosquito Magnet	Peridomiciliary	0.08	2-67_PEC	*Pi. (Pif.) robusta*			+
Ecuador	Pucapamba	Mosquito Magnet	Peridomiciliary	0.08	2-66_PEC	*Pi. (Pif.) robusta*			+
Ecuador	Pucapamba	Mosquito Magnet	Peridomiciliary	0.08	2-65_PEC	*Pi. (Pif.) robusta*			+
Ecuador	Isimanchi	Mini CDC light trap	Intradomiciliary	0.08	2-62_PEC	*Pi. (Pif.) robusta*			+
Ecuador	Chito-Juntas	Protected human bait	Secondary forest	0.25	2-61_PEC	*Pi. (Pif.) robusta*			+
Ecuador	Chito-Juntas	Protected human bait	Secondary forest	0.25	2-44_PEC	*Pi. (Pif.) robusta*	+		+

### Sequence analysis and phylogenetic inference

**Fig 5:**
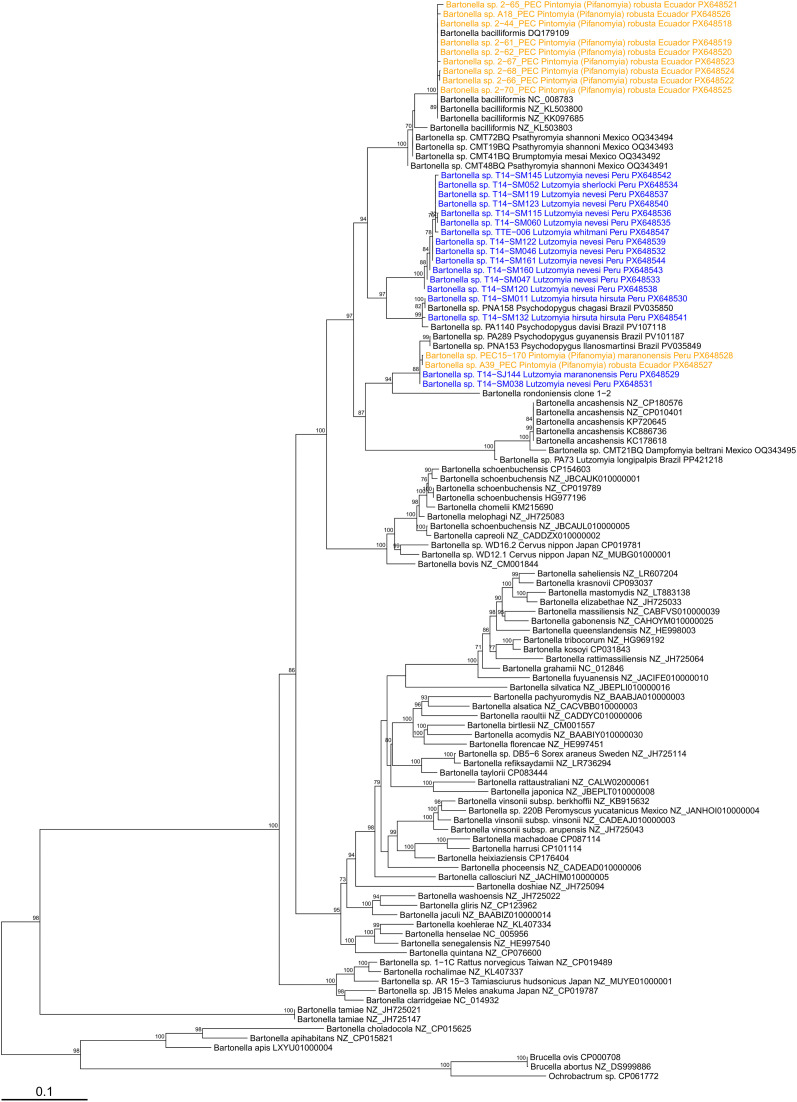
Maximum likelihood phylogenetic tree for *Bartonella* species in phlebotomine sand fly pools based on *gltA* gene sequences. The tree was inferred from a 1290 bp alignment of 114 sequences. The best model of sequence evolution was a generalized time-reversible model with unequal base frequencies and six free rate categories (GTR + F + R4) based on the Bayesian information criterion. *Brucella abortus*, *Brucella ovis*, and *Ochrobactrum* sp. MT180101 were included as outgroups and the tree was rooted at the midpoint. Bootstrap support values ≥70% (out of 1000 iterations) are displayed next to branches and the scale bar indicates substitutions per site. Sequences identified as part of this study are shown in orange while sequences identified by Zorrilla *et al.* (2021) [25] in Peru are shown in blue.

**Fig 6:**
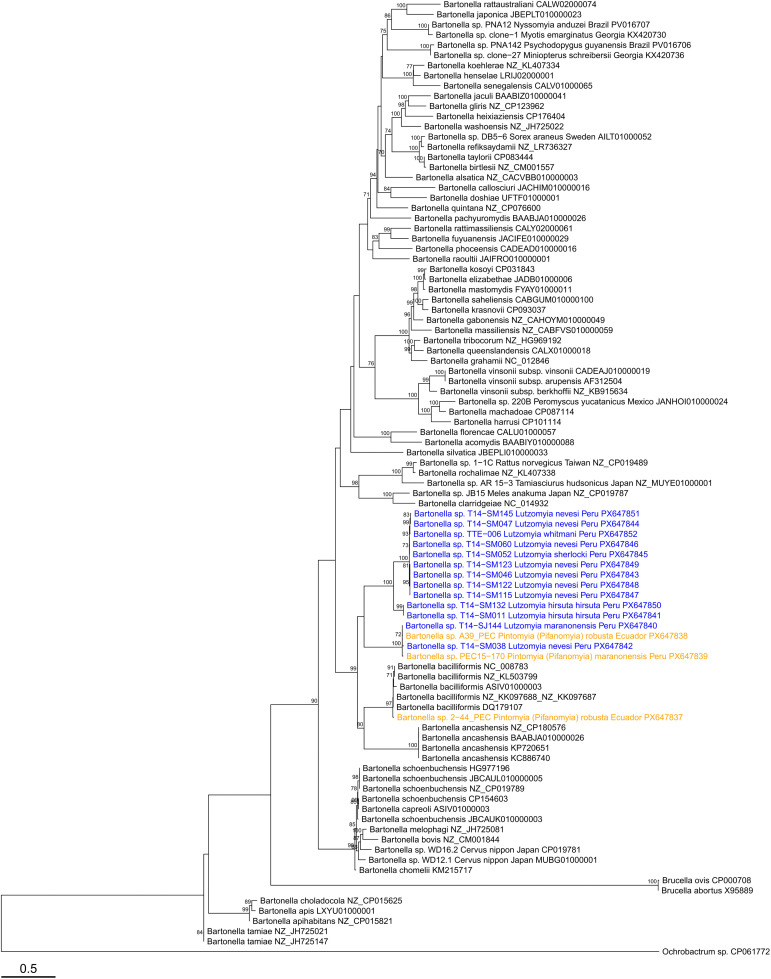
Maximum likelihood phylogenetic tree for *Bartonella* species in phlebotomine sand fly pools based on ITS gene sequences. The tree was inferred from a 1622 bp alignment of 94 sequences (gaps included). The best model of sequence evolution was a transition model with unequal base frequencies and six free rate categories (TIM3 + F + R6) based on the Bayesian information criterion. *Brucella abortus*, *Brucella ovis*, and *Ochrobactrum* sp. MT180101 were included as outgroups and the tree was rooted at the midpoint. Bootstrap support values ≥70% (out of 1000 iterations) are displayed next to branches and the scale bar indicates substitutions per site. Sequences identified as part of this study are shown in orange while sequences identified by Zorrilla *et al.* (2021) [25] in Peru are shown in blue.

**Fig 7:**
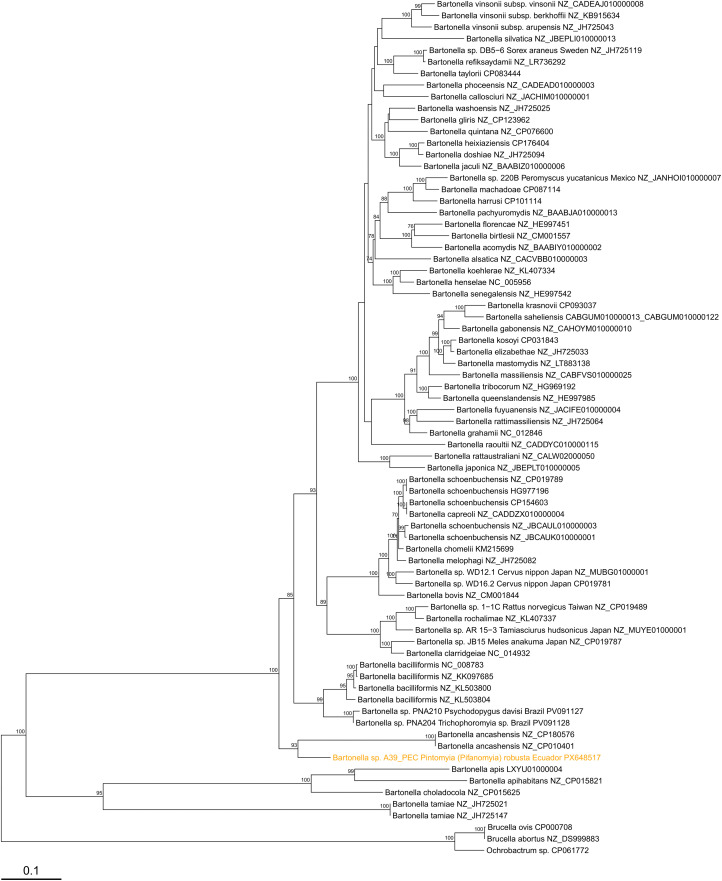
Maximum likelihood phylogenetic tree for *Bartonella* species in phlebotomine sand fly pools based on *nuoG* gene sequences. The tree was inferred from a 2070 bp alignment of 74 sequences. The best model of sequence evolution was a generalized time-reversible model with unequal base frequencies and six free rate categories (GTR + F + R6) based on the Bayesian information criterion. *Brucella abortus*, *Brucella ovis*, and *Ochrobactrum* sp. MT180101 were included as outgroups and the tree was rooted at the midpoint. Bootstrap support values ≥70% (out of 1000 iterations) are displayed next to branches and the scale bar indicates substitutions per site. Sequences identified as part of this study are shown in orange.

A total of 11 amplicons from all positive nested *gltA* PCR products were sequenced (Table 4) and confirmed as *Bartonella* using BLASTn (Table 5); 10 sequences corresponded to *Pi.* (*Pif.*) *robusta* (four from Chito-Juntas, one from Isimanchi, five from Pucapamba, Ecuador) and one sequence to *Pi. (Pif.) maranonensis* (from San Pedro, Namballe, Peru). A phylogenetic tree based on *gltA* gene sequences (Fig 5) showed that nine DNA sequences from Ecuador were closely related to *B. bacilliformis*, showing 0–2 nucleotide differences (99.4–100% sequence identity) compared to the known *B. bacilliformis* strains from Peru (Table 5). This is the first report of *Pi. (Pif.) robusta* with detectable *B. bacilliformis* DNA. Additionally, two DNA sequences, one from *Pi. (Pif.) maranonensis* from Namballe, Peru, and the other one from *Pi. (Pif.) robusta* from Chito-Juntas, Ecuador, were most closely related to DNA sequences previously identified in phlebotomine sand flies from Peru [25], including *Lutzomyia maranonensis* (T14-SJ144) and *Lutzomyia nevesi* (T14-038). Furthermore, these sequences from Peru and Ecuador clustered with *Bartonella* sequences from sand flies in Brazil [42,43], with the closest match available on GenBank being a *Bartonella* sequence amplified from *Psychodopygus llanosmartinsi* in Brazil (accession PV035849; 99.4–99.6% sequence identity). Deeper in the phylogeny, this lineage was related to *Candidatus* B. rondoniensis, sharing 88.1–88.4% sequence identity and forming a clade with 92% bootstrap support. Analysis of ITS sequences confirmed the presence of *B. bacilliformis* (99.3% sequence identity) in sample 2–44_PEC. The other two sequences obtained for this marker, PEC15–170 and A39_PEC, were divergent from known *Bartonella* species and clustered with ITS sequences from *Lu. maranonensis* (T14-SJ144) and *Lu. nevesi* (T14-S038) previously identified in sand flies from Peru [25] (Fig 6). The closest BLASTn match for these sequences was *B. bacilliformis*, sharing only 93.4% sequence identity (Table 5). The single *nuoG* sequence from sample A39_PEC was also divergent from known *Bartonella* species (Fig 7). The closest match via BLASTn was a *Bartonella nuoG* sequence amplified from the bat species *Platyrrhinus helleri* in Peru (91.8% sequence identity; Table 5), though the sequence clustered with *B. ancashensis* in the phylogeny (Fig 7), sharing 87.5% sequence identity with this species and forming a clade with 93% bootstrap support. It is possible that these divergent lineages of ITS and *nuoG* sequences would have clustered with *Bartonella* sequences from other sand fly species tested in Brazil [42,43] or Mexico [41], or with *Candidatus* B. rondoniensis [40], but there were no representative sequences for these gene markers available for comparison from those studies. All *Bartonella* sequences obtained in this study and from the authors’ previous study in Peru [25] have been submitted to GenBank: PX648518–PX648548 (*gltA*), PX647837–PX647852 (ITS), and PX648517 (*nuoG*). GenBank accession numbers for all taxa used in the phylogenetic analyses are provided in S7 Table.

**Table 5:** Results of BLASTn searches for *Bartonella* DNA sequences detected in phlebotomine sand flies. GenBank accession numbers are shown for reference *Bartonella* strains. Sequence identity is shown as a percentage with the number of matching bases over the length of the query sequence shown in parentheses.

Sample ID (accession)	Molecular marker	GenBank reference match (accession)	Sequence length (bp)	Query cover (%)	E-value	Identity (%)
2-44_PEC *Pintomyia* (*Pifanomyia*) *robusta* Ecuador (PX648518)	*gltA*	*B. bacilliformis* strain Bb-6 Peru (OR515766)	348	100	5E-180	100.00
2-61_PEC *Pintomyia* (*Pifanomyia*) *robusta* Ecuador (PX648519)	*gltA*	*B. bacilliformis* strain Bb-6 Peru (OR515766)	355	100	0	100.00
2-62_PEC *Pintomyia* (*Pifanomyia*) *robusta* Ecuador (PX648520)	*gltA*	*B. bacilliformis* strain Bb-6 Peru (OR515766)	355	100	0	100.00
2-65_PEC *Pintomyia* (*Pifanomyia*) *robusta* Ecuador (PX648521)	*gltA*	*B. bacilliformis* strain Bb-6 Peru (OR515766)	351	100	2E-178	99.43
2-66_PEC *Pintomyia* (*Pifanomyia*) *robusta* Ecuador (PX648522)	*gltA*	*B. bacilliformis* strain Bb-6 Peru (OR515766)	293	100	2E-149	100.00
2-67_PEC *Pintomyia* (*Pifanomyia*) *robusta* Ecuador (PX648523)	*gltA*	*B. bacilliformis* strain Bb-6 Peru (OR515766)	359	100	0	99.72
2-68_PEC *Pintomyia* (*Pifanomyia*) *robusta* Ecuador (PX648524)	*gltA*	*B. bacilliformis* strain Bb-6 Peru (OR515766)	360	100	0	99.72
2-70_PEC *Pintomyia* (*Pifanomyia*) *robusta* Ecuador (PX648525)	*gltA*	*B. bacilliformis* strain Bb-6 Peru (OR515766)	360	100	0	100.00
A18_PEC *Pintomyia* (*Pifanomyia*) *robusta* Ecuador (PX648526)	*gltA*	*B. bacilliformis* strain Bb-6 Peru (OR515766)	353	100	0	99.72
A39_PEC *Pintomyia* (*Pifanomyia*) *robusta* Ecuador (PX648527)	*gltA*	*Bartonella* sp. clone PNA153 *Psychodopygus llanosmartinsi* Brazil (PV035849)	271	100	1E-135	99.63
PEC15–170 *Pintomyia* (*Pifanomyia*) *maranonensis* Peru (PX648528)	*gltA*	*Bartonella* sp. clone PNA153 *Psychodopygus llanosmartinsi* Brazil (PV035849)	353	99	2E-178	99.43
2-44_PEC *Pintomyia* (*Pifanomyia*) *robusta* Ecuador (PX647837)	ITS	*B. bacilliformis* strain CON600–01 Peru (AJ422179)	417	100	0	99.28
A39_PEC *Pintomyia* (*Pifanomyia*) *robusta* Ecuador (PX647838)	ITS	*B. bacilliformis* strain ER-Yal Peru (AJ422178)	465	39	2E-66	93.41
PEC15–170 *Pintomyia* (*Pifanomyia*) *maranonensis* Peru (PX647839)	ITS	*B. bacilliformis* strain ER-Yal Peru (AJ422178)	500	45	2E-66	93.41
A39_PEC *Pintomyia* (*Pifanomyia*) *robusta* Ecuador (PX648517)	*nuoG*	*Bartonella* sp. strain PL B43033 *Platyrrhinus helleri* Peru (MN270106)	256	100	5E-94	91.80

## Discussion

The transmission dynamics of both leishmaniasis and human bartonellosis are complex, with numerous phlebotomine sand fly vectors implicated in transmission, as well as pathogen, human, and environmental factors playing a role. These neglected diseases are serious threats to public health, particularly in low-income communities with limited health care access. Additionally, service members deployed to endemic areas can be targets for exposure to infected phlebotomine sand fly bites, thus increasing disease risk, which can necessitate medical evacuations, reduce operational readiness, and disrupt missions [49].

One of the most critical components to understanding and preventing transmission of these co-circulating human diseases is identifying which phlebotomine sand fly species harbor and transmit the pathogens in specific ecological environments. The Peru-Ecuador border covers an extensive area, with unique ecological features on each side. This region extends from the high jungle rainforest across the Andean valleys and Pacific coast foothills. Our study sites in Namballe/San Ignacio (Peru) and Zamora-Chinchipe (Ecuador) are located in the middle of the Chinchipe River basin with mountains, slopes, and valleys spanning Andean and rainforest habitats. Three phlebotomine sand fly species were identified in this region: *Pi. (Pif.) robusta, Pi. (Pif.) maranonensis* and *Lu. (Hel.) castanea*. Peridomestic coffee plantations located between human dwellings and the forest edge represent suitable habitats for these phlebotomine sand fly species. In contrast, the ecological features of the coastal region along the Ecuador-Peru border influence phlebotomine sand fly fauna composition, having recorded 15 and 11 species in the Ecuadorian and Peruvian sides, respectively, with only two species, *Lu. (Trl.) gomezi* and *Pa. (Psa.) shannoni*, present at both sides. The Peruvian northern coastal region and western valleys are dominated by seasonally dry tropical forest and xerophytic scrubland, with marked seasonality in rainfall, higher temperatures, and more heterogeneous land-use patterns related to agriculture and livestock. In contrast, the adjacent Ecuadorian coastal region harbors remnants of wetter tropical forest, higher annual rainfall, and vegetation with greater structural complexity [23,24,50,51]. These ecological differences may explain the variation in phlebotomine sand fly species diversity, despite the geographic proximity of both regions [7,19,20,52].

In the 1990s, *Pi. (Pif.) robusta* was reported as the most plausible vector of leishmaniasis from the Ecuadorian side of the border [7]; however, it was never found naturally infected with either *Leishmania* or *Bartonella* spp. The results of this study revealed that *Pi. (Pif.) robusta* specimens collected from Chito-Juntas, Pucapamba, and Ishimanchi in Ecuador were positive for *B. bacilliformis* DNA, which, to the best of our knowledge, represents the first evidence of pathogen infection of this phlebotomine sand fly species. Another common phlebotomine sand fly species on the Ecuador-Peru border is *Pi. (Pif) maranonensis*, which has been found infected with *B. bacilliformis* and *Leishmania* parasites in Peru [9,18]. Both species, *Pi. (Pif.) robusta* and *Pi. (Pif.) maranonensis* are very aggressive anthropophilic species of sylvatic origin that have become adapted to the domestic environment, seeking blood meals both indoors and outdoors [7]. With the reporting of Carrion’s disease in 2022 for the first time since 1996 in Ecuador [13], and the outbreak in Peru in 2013 [17], we provide evidence that implicates *Pi. (Pif.) robusta* in the transmission of Carrion’s disease at the Ecuador-Peru border.

Furthermore, our phylogenetic analyses revealed a close genetic relationship between the *B. bacilliformis* samples detected in sand flies from Ecuador and the isolates known to circulate in Peru. This suggests that the *B. bacilliformis* populations in both countries share a relatively recent common ancestor. The high similarity could also indicate a geographic or historical connection between the bacterial populations. The detection of highly related bacterial species in two geographically close locations may indicate that the disease is spreading between these two regions. These findings could have important implications for the epidemiology and understanding of the evolution of *B. bacilliformis* in the region, and the study of Carrion’s disease transmission in Peru and Ecuador.

In this study, *Candidatus* B. rondoniensis-like DNA was also detected in *Pi. (Pif.) robusta* from Chito-Juntas, Zamora-Chinchipe, Ecuador, and in *Pi. (Pif.) maranonensis* from San Pedro, Namballe district, Peru, based on *gltA* sequences. Recently, *Pi. (Pif.) maranonensis* collected from San Jose de Lourdes district, San Ignacio, Cajamarca, were found harboring *Candidatus* B. rondoniensis*-*like sequences [25]. The *nuoG* sequence detected in a single sample (A39-PEC) was highly divergent from currently available *Bartonella* reference sequences. Phylogenetic analysis placed this sequence on a long branch attached to the *Bartonella ancashensis* clade, while this same sample was part of a larger group of sand fly sequences that clustered within the wider *B. bacilliformis*/*B. ancashensis* clade according to ITS and *gltA*. This apparent discrepancy likely reflects marker-specific resolution, limited representation of *nuoG* sequences from *Bartonella* detected in sand flies on GenBank, and the complex nature of arthropod-derived DNA, where low pathogen load and abundant host and microbiome DNA may influence amplification and phylogenetic signal [39,53]. Therefore, this result is best interpreted as reflecting dataset and molecular marker limitations rather than underestimation of *B. bacilliformis* or *B. ancashensis*. In the Brazilian Amazon region, *Bartonella* sequences detected in *Psychodopygus guyanensis* and *Psychodopygus llanosmartinsi*, were previously reported as clustering within the *B. bacilliformis*/*B. ancashensis* clade based on *gltA* sequences [42,43]. However, when these sequences were included in the present phylogenetic analysis along with additional references, they clustered closer to *Candidatus* B. rondoniensis, but still within the *B. bacilliformis*/*B. ancashensis* clade (Fig 5). Accordingly, these sequences, along with the sand fly sequences reported here from Ecuador and Peru, are best interpreted as belonging to a *Candidatus* B. rondoniensis-like lineage within the broader *B. bacilliformis/B. ancashensis* clade. Sequencing of additional genetic markers and multi-locus phylogenetic analysis is in progress for some of these samples and will aid in resolving some of the uncertainty around the phylogenetic position of these new sand fly-associated lineages. *Candidatus* B. rondoniensis was first found in triatomine bugs from French Guiana [40], and it is unknown if this lineage of *Bartonella* is pathogenic for humans and/or animals. The province of Loja in Southern Ecuador and the states of Cajamarca and Amazonas in northern Peru are endemic areas for Chagas disease [54,55], yet there are no reports on triatomine bugs naturally infected with *Bartonella* spp. at these border regions.

A limitation of this study is that amplification of an endogenous insect gene, COI, was not performed for all sand fly DNA extracts as an internal control for PCR performance. COI amplification was conducted only for a subset of specimens for DNA barcoding purposes and was not systematic across all samples. Consequently, although negative controls were consistently included and no evidence of contamination was detected, the possibility of false-negative results due to DNA degradation or PCR inhibition cannot be completely excluded. This limitation should be considered when interpreting the apparent absence of *Bartonella* DNA in the samples analyzed and the estimated prevalence values, which are likely underestimates.

Leishmaniasis-endemic areas are widely distributed along the Ecuador-Peru border where, besides *Pi. (Pif.) robusta* and *Pi. (Pif.) maranonensis*, other potential vectors including *Ny. trapidoi*, *Pi. (Pif.) serrana*, *Lu. (Trl.) gomezi*, and *Lu. (Hel.) hartmanni* in Ecuador and *Lu. (Hel.) ayacuchensis*, and *Mi. (Mic.) cayennensis cayennensis* in Peru have been recorded. However, no *Leishmania* DNA was detected in phlebotomine sand flies collected in this study, which could be linked to the temporary and complex pattern of leishmaniasis transmission influenced by weather conditions, phlebotomine sand fly population densities, and the presence of reservoirs. Additionally, the infection rate of *Lutzomyia* with *Leishmania* has been shown to be very low in the New World, in some cases < 1% [27,56].

The Ecuador-Peru border has comparable ecological features comprising a similar and not very diverse phlebotomine sand fly fauna with *Pi. (Pif.) robusta, Pi. (Pif.) maranonensis*, and *Lu. (Hel.) castanea* being the most abundant [7]. Local inhabitants from both countries travel across the border between the cities of Namballe and San Ignacio (Peru) and Zumba (Ecuador) through a main road that connects the Amazonian region of both countries. Despite the regular flow of people and similar ecologies and phlebotomine sand fly fauna, the diverging bartonellosis reporting patterns in Peru and Ecuador may be due to human-related factors including: (i) the different distribution of inhabitants along the border in each country, (ii) house construction based on adobe and dried palm leaves in Peruvian houses close to the border versus houses made of bricks and cement on the Ecuadorian side, and (iii) the road networks in each country, with a paved highway on the Peruvian side versus a secondary road (at the time of the study) on the Ecuadorian side connecting towns such as Pucapamba and Zumba. Houses built with adobe could lead to more phlebotomine sand fly breeding sites and house construction has been found to be a key factor in the transmission of other vector-borne diseases, such as Chagas in southern Ecuador [57], and reduced infrastructure and urbanization could lead to disease underreporting due to inadequate medical facilities and poor disease surveillance.

Effective entomological surveillance relies on choosing the appropriate collection methods that are best for a successful collection and detection of infection in phlebotomine sand fly populations. Several traps and methods have been evaluated for the collection of mosquito vectors, including the CDC Miniature light traps, Mosquito Magnet and protected human bait [58–61], which have proven to be effective for phlebotomine sand flies and sand fly-borne pathogen surveillance [25,62]. The collections carried out during this study suggest that resting landing collections with protected human bait remains an effective collecting method, especially for anthropophilic phlebotomine sand fly species that may not be adequately detected using CDC light traps.

For molecular detection of *Leishmania* and *Bartonella* DNA, we used conventional PCR, a widely validated approach that allows downstream sequencing and species-level characterization at a lower cost when processing large numbers of samples. However, several studies have demonstrated that quantitative PCR (qPCR), digital PCR (dPCR), and droplet digital PCR (ddPCR) provide higher analytical sensitivity, particularly in samples with low bacterial loads, such as arthropod vectors. Reported detection rates for *Bartonella* are consistently higher using dPCR compared with qPCR and conventional PCR, reflecting its ability to detect low-copy targets and reduce the impact of PCR inhibitors commonly present in arthropod-derived DNA. Andre *et al*. (2015) [63] reported that qPCR assay detected *Bartonella* DNA in 46 out of 151 sampled Brazilian domiciled cats, while conventional PCR detected only 39.1% of these positive samples. In another study, Calchi *et al*. (2025) [64] demonstrated significantly higher sensitivity of dPCR compared to qPCR for *Bartonella* DNA detection in Brazilian wild animals, showing a 35.26% positivity rate with dPCR, while qPCR detected only 10.66%. For *Leishmania* DNA detection, Ramirez *et al*. (2019) [65] reported that the limit of detection for ddPCR was 100 parasites/mL compared to 1 parasite/mL for qPCR and concluded that the qPCR platform is more suitable for clinical diagnostic purposes. In contrast, Alvaro *et al*. (2025) [66] found high sensitivity of dPCR, capable of detecting one *Leishmania* parasite cell in a reaction. Despite these advantages, dPCR remains limited by higher costs, lower accessibility, and the inability to generate amplicons suitable for sequencing, which constrains taxonomic and phylogenetic analyses. Therefore, while qPCR and dPCR are valuable tools for improving *Bartonella* and *Leishmania* detection and estimating infection prevalence, conventional PCR remains appropriate for ecological and phylogenetic studies where sequence information is required. Further surveillance efforts focused on phlebotomine sand fly vectors should incorporate these methodologies and assess improvements in pathogen detection.

This study sheds light on phlebotomine sand fly species associated with pathogen transmission in the understudied Ecuador-Peru border and highlights the role of *Pi. (Pif.) robusta* in the transmission of human bartonellosis, a neglected disease in Andean countries. In addition, we report the presence of a new *Bartonella* lineage circulating in this region, with more studies needed to understand what role, if any, this bacterium plays in human disease. To best understand the complex nature of these pathogens, a One Health approach is needed to clarify the epidemiological factors shaping human bartonellosis and leishmaniasis transmission dynamics at the Ecuador-Peru border.

## Supporting information

S1 Table Phlebotomine sand fly species collected and identified in indoor, outdoor, and forest areas in five sites in southern Ecuador during 2015–2017. (XLSX)

S2 Table Phlebotomine sand fly species collected and identified by collection trap and sex from five sites in southern Ecuador in 2015–2017. (XLSX)

S3 Table Phlebotomine sand fly species collected per hour/trap from five sites in southern Ecuador in 2015–2017. (XLSX)

S4 Table Phlebotomine sand fly species collected and identified indoors and outdoors in four sites in northern Peru during 2015–2016. (XLSX)

S5 Table Phlebotomine sand fly species collected and identified by collection trap and sex from four sites in northern Peru in 2015–2016. (XLSX)

S6 Table Phlebotomine sand fly species collected per hour/trap from four sites in northern Peru in 2015–2016. (XLSX)

S7 Table GenBank accession numbers for all bacterial taxa used in the phylogenetic analyses. (XLSX)

S1 Text PCR conditions for *Bartonella* and *Leishmania* DNA detection in phlebotomine sand flies. (DOCX)
