# Supplementary material for: Phlebotomine sand fly fauna characterization and Bartonella bacilliformis DNA detection in Pintomyia (Pifanomyia) robusta at the Ecuador-Peru frontier
Source: PLoS Negl Trop Dis. 2026 May 13;20(5):e0014288. doi: 10.1371/journal.pntd.0014288 (PMC13170852; doi:10.1371/journal.pntd.0014288)
Supplement: S1 Text — (DOCX) [file pntd.0014288.s008.docx]

**S1 Text - PCR conditions for *Bartonella* and *Leishmania* DNA detection in phlebotomine sand flies**

**Primers used for detection of *Leishmania* and *Bartonella* DNA**

| **Primer** | **Locus** | **5'-sequence-3'** | **Size of PCR Product** | **Protocol** | **Author** |
| --- | --- | --- | --- | --- | --- |
| ***Bartonella*** |  |  |  |  |  |
| ITS 325S | 16S–23S internal transcribed spacer | CTTCAGATGATGATCCCAAGCCTTTTGGCG | 400-600bp | PCR | [1] |
| ITS 1100AS | 16S–23S internal transcribed spacer | GAACCGACGACCCCCTGCTTGCAAAGCA |  |  |  |
| gltA 443F | *gltA* citrate synthase gene | GCTATGTCTGCATTCTATCA | 767bp | Nested-PCR, first | [2] |
| gltA 1210R | *gltA* citrate synthase gene | GATCYTCAATCATTTCTTTCCA |  |  |  |
| gltA BhCS.781p | *gltA* citrate synthase gene | GGGGACCAGCTCATGGTGG | 380-400 bp | Nested-PCR, second | [3] |
| gltA BhCS.1137n | *gltA* citrate synthase gene | AAATGCAAAAAGAACAGTAAACA |  |  |  |
| nuoG-F | NADH dehydrogenase gamma subunit | GGCGTGATTGTTCTCGTTA | 380-400 bp | PCR | [39] |
| nuoG-R | NADH dehydrogenase gamma subunit | CACGACCACGGCTATCAAT |  |  |  |
|  |  |  |  |  |  |
| ***Leishmania*** |  |  |  |  |  |
| L.MC-1S | kinetoplast DNA | CTRGGGGTTGGTGTAAAATAG | 700bp | PCR | [34] |
| L.MC-1R | kinetoplast DNA | TWTGAACGGGRTTTCTG |  |  |  |
| L.cyt-S | cytochrome b gene | GGTGTAGGTTTTAGTYTAGG | 730-850bp | Nested-PCR, first | [34] |
| L.cyt-R | cytochrome b gene | CTACAATAAACAAATCATAATATRCAATT |  |  |  |
| L.cyt-AS | cytochrome b gene | GCGGAGAGRARGAAAAGGC | 1070-1080bp | Nested-PCR, second | [4,5] |
| L.cyt-ASR | cytochrome b gene | CCACTCATAAATATACTATA |  |  |  |

**Molecular detection of *Leishmania* DNA**

The conventional PCR reaction was carried out in a final volume of 25 µL with 1 µL of DNA template, using MgCl_2_ at a 1.5mM final concentration, dNTPs at a 0.2 mM final concentration and 1.25 units of GoTaq Flexi DNA polymerase (Promega, Madison, WI). After an initial denaturation at 95°C for 5 minutes, the PCR amplification was performed with 30 cycles of denaturation (95°C for one minute), annealing (55°C for one minute), and polymerization (72°C for one minute), with a final extension at 72°C for 10 minutes^34^. *Leishmania* species were identified in positive samples using the partial cytochrome *b* gene (between 730-850bp) amplified with a nested PCR^4,5^. The first PCR amplification was performed using L.cyt-AS and L.cyt-AR primers and 2µL of DNA. The second PCR amplification was performed using L.cyt-S and L.cyt-R primers and 1µL of the first PCR. The PCR conditions of both PCRs were the same as the kDNA PCR.

**Molecular detection of *Bartonella* DNA**

**ITS PCR.** The PCR was carried out in a volume of 25 µL with 5 µL of DNA template, using ITS 325S and ITS 1100AS primers at a 0.4 µM final concentration^1^ and Platinum High Fidelity Master Mix PCR solution (Invitrogen, Carlsbad, CA). After initial denaturation at 95°C for 3 minutes, the PCR was performed with 55 cycles of denaturation (95°C for 30 seconds), annealing (66°C for 30 seconds), polymerization (72°C for 30 seconds), and final extension at 72°C for 7 minutes.

**Nested *gltA* PCR**. The first amplification of *gltA* nested PCR was performed using 443F and 1210R primers at a 0.4 µM final concentration^2^ and Platinum High Fidelity Master Mix PCR solution (Invitrogen, Carlsbad, CA), with the following PCR conditions: initial denaturation at 95°C for 5 minutes followed by 20 cycles of denaturation (95°C for 30 seconds), annealing (48°C for 30 seconds), polymerization (72°C for 2 minutes), and final extension at 72°C for 7 minutes. The Bhcs.781p and Bhcs.1137n primers at a 0.4 µM final concentration^3^ were used to second PCR amplification with the following PCR conditions: initial denaturation at 95°C for 5 minutes followed by 40 cycles of denaturation (95°C for 30 seconds), annealing (55°C for 30 seconds), polymerization (72°C for 1 minute), and final extension at 72°C for 7 minutes.

**nuoG PCR**. The PCR was carried out at a volume of 25 µL with 5 µL of DNA template, using nuoG-F and nuoG-R primers^39^ at a 0.4 µM final concentration and Platinum High Fidelity Master Mix PCR solution (Invitrogen, Carlsbad, CA). After initial denaturation at 95°C for 5 minutes, the PCR was performed with 37 cycles of denaturation (95°C for 30 seconds), annealing (66°C for 30 seconds), polymerization (72°C for 1 minute), and final extension at 72°C for 10 minutes.

**References**

1. Billeter S, Miller M, Breitschwerdt E, Levy M. Detection of Two *Bartonella tamiae*-Like Sequences in *Amblyomma americanum* (Acari: Ixodidae) Using 16S-23S Intergenic Spacer Region-Specific Primers. J Med Entomol. 2008b. 45(1):176-179.
2. Billeter S, Caceres A, Gonzales J, Luna D, Kosoy M. Molecular detection of *Bartonella* species in ticks from Peru. J Med Entomol. 2011; 48(6):1257–60. https://doi.org/10.1603/me10240.
3. Norman A, Regnery R, Jameson P, Greene C, Krause D. Differentiation of *Bartonella*-like isolates at the species level by PCR-Restriction Fragment length polymorphism in the Citrate Synthase Gene. J Clin Microbiol. 1995; 33(7):1797–1803. https://doi.org/10.1128/jcm.33.7.1797-1803.1995
4. Kato H, Cáceres AG, Mimori T, et al. Use of FTA cards for direct sampling of patients’ lesions in the ecological study of cutaneous leishmaniasis. J Clin Microbiol. 2010; 48:3661–5.
5. Kato H, Calvopiña M, Criollo H, Hashiguchi Y. First human cases of *Leishmania (Viannia) naiffi* infection in Ecuador and identification of its suspected vector species. Acta Tropica. 2013; 128: 710–713. https://doi.org/10.1016/j.actatropica.2013.09.001 PMID: 24044975
